# Editorial

**Published:** 1987-02

**Authors:** 


### Editorial

### THE JOURNAL IN 1987

We may well look back on 1986 as a golden age. The firm whose sponsorship encouraged Hermiston to propose a partnership with us and who funded us in 1986 has withdrawn. Pfizer have stepped in and will contribute very substantially this year, our parent society will play its part but there will have to be two changes. We shall revert to our former quarterly issues and the journal will be distributed free only to those West Country doctors who request it. This latter measure is in any case a reasonable one. Doctors in the West Country have been able to read the journal for a year and can now decide whether they want to go on getting it. To send it indefi nitely to those who don't is surely wasteful. Former subscribers, members of the Bristol Medico-Chirurgical Society and of those Societies who contribute abstracts of their meetings will continue to receive the journal without application.

### REGIONAL REPRESENTATION ON THE JOURNAL

We are glad to be able to tell our readers that our aspiration to become a genuine MEDICAL JOURNAL OF THE SOUTH WEST has made progress with the addition of Professor Pereira Gray of Exeter, and Mr Colin Teasdale of Plymouth to our panel of correspondents. They join Dr Adam of Taunton who has been contributing during the past year, this means that besides the area representation the branches of general practice, general surgery and pathology also have a voice, we hope next to find a physician who will speak for Cornwall.

### SALUTE TO A NEW JOURNAL

Mr David Leaper, Senior Lecturer in the Bristol University Department of Surgery has taken the initiative in starting a new journal, the first in Britain to be devoted to the science of surgery, SURGICAL RESEARCH COMMUNICATIONS. This will be an international journal with an international editorial board and will certainly play an important part in advancing research in surgery and disseminating knowledge of each other's work among researchers. Such a journal is now much needed, the 'Waiting List' for publication of important research is too long and is getting longer. Mr Leaper is following in the footsteps of another Bristol surgeon, Ernest Hey Groves who founded the BRITISH JOURNAL OF SURGERY in 1913 and who was its editor for 27 years. It is still one of the best journals of surgery in any language. We wish Mr Leaper a similar success and a similar term of office.

### NEW HOSPITAL BUILDING IN THE SOUTH WEST

In our last number we reported on the new hospital at Weston-super-mare and the new Bristol Eye Hospital both recently opened, also the new hospital at Musgrove Park, Taunton to be opened in February. In this number we report on the redevelopment of Frenchay Hospital. Work on a ten-ward block with Burns Unit and Day Centre for the Elderly has already begun. Approval of the plan for a new Orthopoedic Hospital at Southmead was given in 1985. This is a period of hospital building in the South West which must be almost unprecedented. *It is to be hoped that this* payment to Paul has not robbed Peter of some of his expectations.

